# Photoacoustic based evaluation of viscoelastic properties of Gram-negative and Gram-positive bacterial colonies

**DOI:** 10.1038/s41598-023-41663-8

**Published:** 2023-09-05

**Authors:** Zahra Hosseindokht, Mohammadreza Kolahdouz, Bahareh Hajikhani, Pezhman Sasanpour

**Affiliations:** 1 School of Electrical and Computer Engineering, College of Engineering, University of Tehran, Tehran, Iran; 2 Department of Microbiology, School of Medicine, Shahid Beheshti University of Medical Sciences, Tehran, Iran; 3 Department of Medical Physics and Biomedical Engineering, School of Medicine, Shahid Beheshti University of Medical Sciences, Tehran, Iran

**Keywords:** Biophotonics, Photoacoustics, Bacteriology

## Abstract

Mechanical properties of bacterial colonies are crucial considering both addressing their pathogenic effects and exploring their potential applications. Viscoelasticity is a key mechanical property with major impacts on the cell shapes and functions, which reflects the information about the cell envelope constituents. Hereby, we have proposed the application of photoacoustic viscoelasticity (PAVE) for studying the rheological properties of bacterial colonies. In this regard, we employed an intensity-modulated laser beam as the excitation source followed by the phase delay measurement between the generated PA signal and the reference for the characterization of colonies of two different types of Gram-positive and Gram-negative bacteria. The results of our study show that the colony of *Staphylococcus aureus* as Gram-positive bacteria has a significantly higher viscoelasticity ratio compared to that value for *Acinetobacter baumannii* as Gram-negative bacteria (77% difference). This may be due to the differing cell envelope structure between the two species, but we cannot rule out effects of biofilm formation in the colonies. Furthermore, a lumped model has been provided for the mechanical properties of bacterial colonies.

## Introduction

Cell mechanical properties play crucial roles in various cell functions, including cell growth and division^1,2^. Viscoelasticity as an important mechanical specification of the cells can be considered as a parameter for monitoring physiological function, pathological state, and disease diagnosis^3,4^. Biological cells consist of some constituents with different mechanical roles, for example, the cytoskeleton is relatively rigid, while the cell membrane can be assumed as a viscoelastic component^5,6^.

The cell envelope, which incorporates a range of biological molecules not only must be rigid to maintain positive turgor pressure and the shape of the cell but also must be flexible to allow the transport of nutrients and waste into and out of the cell and the growth and division of the cell^7^. Bacteria are ubiquitous single-celled microorganisms with no nucleus membrane that are responsible for the decomposition and rearrangement of materials in the biological world. They have been known as simple organisms while possessing complex cell envelopes, making them survive in an unpredictable and hostile environment^8–10^. Generally, bacteria can be classified into two groups Gram-positive and Gram-negative according to their envelope structural organization. The envelope of Gram-negative cells is composed of two lipid bilayers separated by periplasm, which contains a thin peptidoglycan layer with 3–8 nm thickness, while the Gram-positive envelope consists of a thick peptidoglycan layer with 20–80 nm thickness, the periplasm, and a plasma membrane^10–12^.

There are different techniques to measure the viscoelasticity of the cells. Tweezing methods in which shear stresses or pressure gradients are applied in suspension and the cell deformation is measured. The tweezers can be categorized as optical tweezers^13–16^, acoustic tweezers^17^, magnetic tweezers^18,19^, micropipette aspiration^20,21^ and deformability cytometry^22–24^. These approaches mainly suffer from being invasive and are subjected to non-linear effects^13,25^. Another technique for measuring the viscoelasticity of a cell is based on atomic force microscopy (AFM) in which cell indentation or height can be measured by applying external force^5,26–30^. By the way, there is a possibility of inducing certain damage by mechanical loading. Furthermore, the difficulties in technical implementation limit its application.

The photoacoustic (PA) technique as a hybrid fast-growing imaging technology, which provides high-resolution sensing of optical contrast beyond the optical transport mean free path, has a wide range of applications including diagnosis of disease^31–34^ and monitoring of pathophysiological states at different levels^35–38^, image-guided drug delivery^39–43^ and preclinical diagnosis^44–49^. In recent years, there have been various modalities developed for PA imaging systems including PA microscopy (PAM)^29,50–53^, PA tomography (PAT)^54–57^ and PA endoscopy (PAE)^58–62^. Although conventional PA systems were designed to measure optical absorption, mechanical properties such as viscoelasticity can be extracted by PA viscoelasticity (PAVE) as well^63^. In this regard, the phase delay between the excited light and generated PA signal could be measured for the calculation of the viscosity-elasticity ratio of the sample in PAVE. PAVE has various advantages compared to conventional methods such as being noninvasive, low cost and needless to extrinsic mechanical loading. PAVE has been utilized for tumor detection^63^, assessment of arterial plaque^64^, cirrhosis detection^65^, identification of esophageal disease^66^ and acute hepatitis detection^67^. Although there are various methods for measuring viscoelasticity such as AFM, tweezers, and other mentioned techniques and each one has its own advantages, considering the required length scale for bacterial colonies, PAVE is the best method not only due to being low-cost and non-destructive but also because of the capability of providing depth resolution, non-operator dependency, linearity and minimum sample preparation requirement.

In the present study, we have employed a PAVE system for the investigation of the viscoelasticity of bacteria in the colony scale. In this regard, the analysis has been performed on the *Acinetobacter baumannii* colony as a Gram-negative bacteria and the *Staphylococcus aureus *colony as a Gram-positive bacteria. The system capability and accuracy have been verified by gelatin phantoms. In addition, a mechanical model has been suggested for colonies of bacteria based on the Kelvin-Voigt model.

## Results

### PAVE of gelatin phantoms

**Figure 1:**
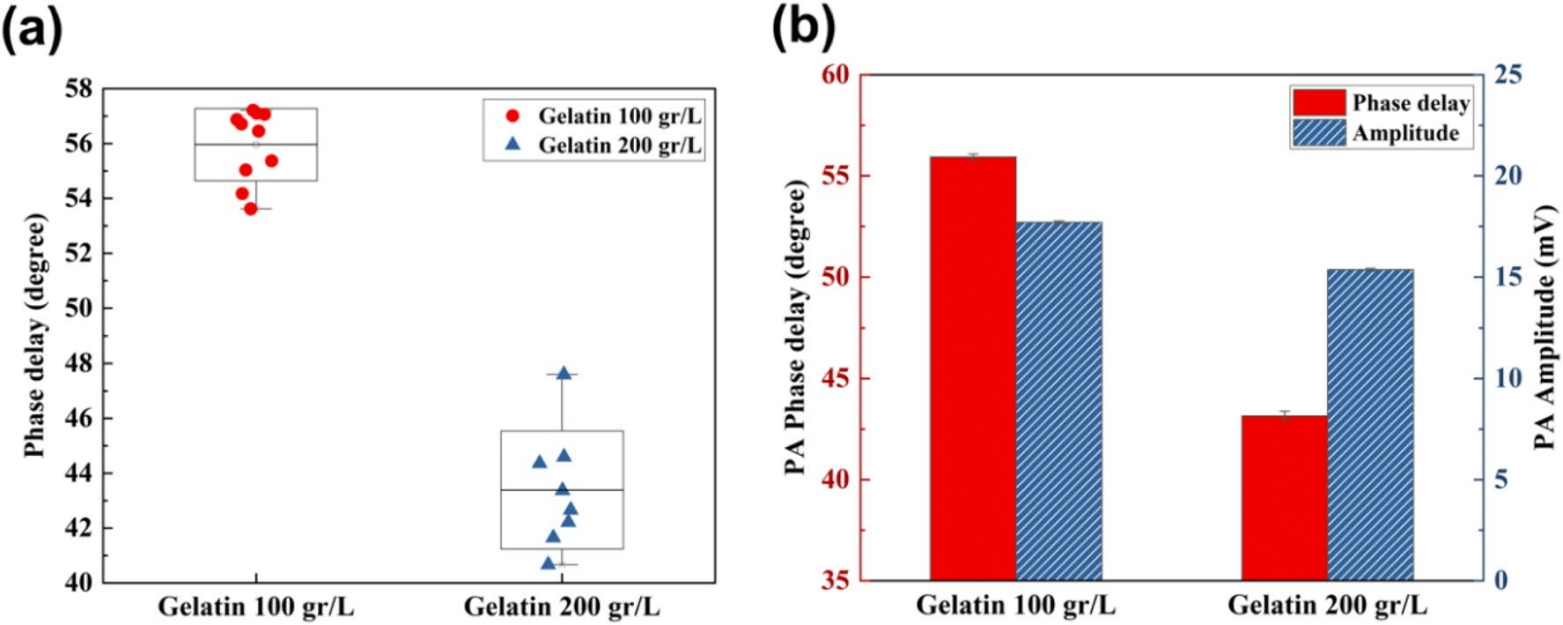
(**a**) Scatter plot of PA phase delay. (**b**) PA amplitude and phase delay of gelatin samples.

Gelatin samples with 100 g/L and 200 g/L concentrations were prepared and the phase delay of the generated PA signals after laser irradiation was calculated. Figure 1a shows the scatter plot of the measured phase delay at different spots of the sample. As expected, the phase delay of the sample with a higher density is lower compared to the sample with less gelatin concentration. In order to show that mechanical properties are mostly reflected in the phase delay (not the PA amplitude), measured values of signal amplitude and phase delay of gelatine samples are shown in Fig. 1b. By doubling the concentration of sample, PA amplitude changed only 2.3 mV (from 17.7 mV for the sample with 100 g/L concentration to 15.4 mV for the gelatine sample with 200 g/L concentration). However, the phase delay had a substantial reduction from 55.93° to 43.15° by doubling the sample density.

### PAVE of biological tissues

**Figure 2:**
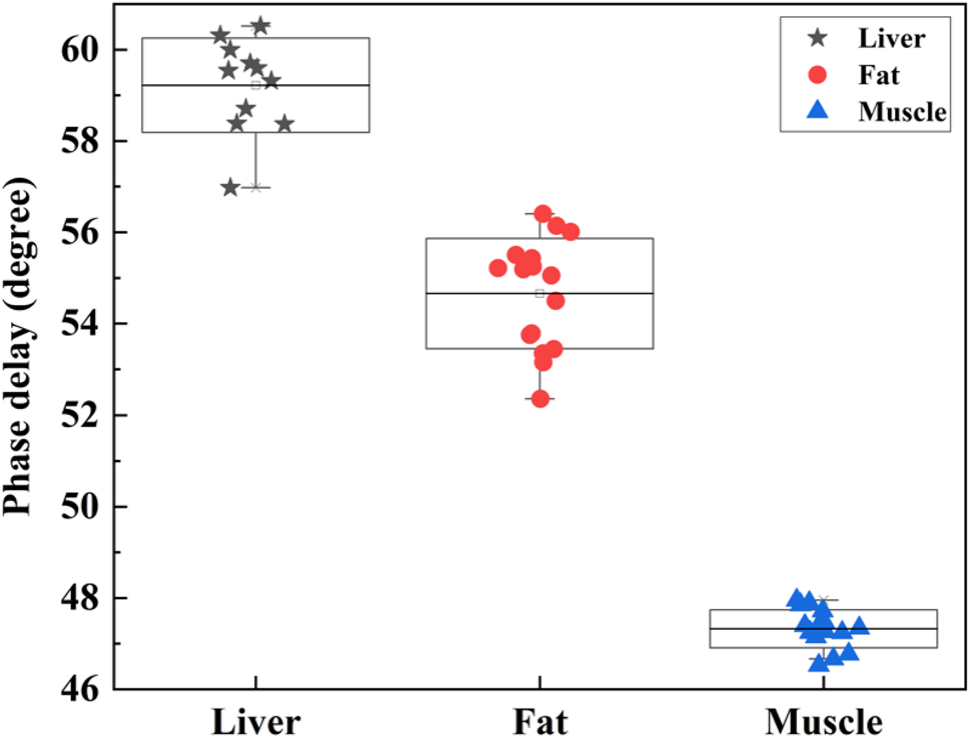
Scatter plot of PA phase delay of chicken tissues.

To verify the system capability, the averages of measured values of PA phase delay of the liver, fat and muscle of chicken are represented in Fig. 2. This graph shows the scatter plot of the phase delay at different spots of each sample. It can be seen that chicken tissues can be distinguished completely based on the phase delay of PA signals. It is good to be noted that muscle has the highest stiffness among these tissues and fat is stiffer compared to the liver.

### PAVE of bacterial colonies

**Figure 3:**
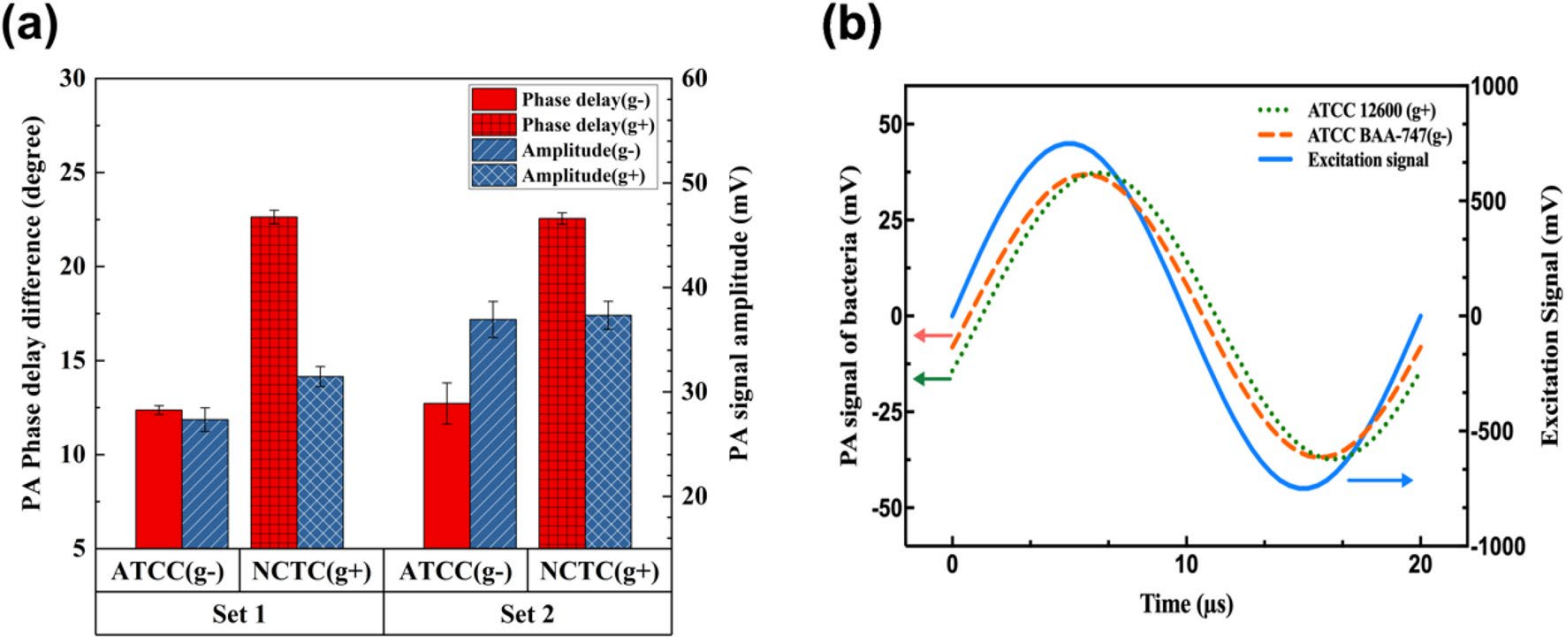
(**a**) Bar chart of PA signals of bacteria. (**b**) Sine wave representation of the PA signal of the second set.

Two sets of cultured bacteria each of which included *Acinetobacter* *baumannii* and *Staphylococcus aureus* were prepared and cut along, and then the samples were put on top of the transducer, which was covered by a thin layer of ultrasound gel as the coupling media. To minimize the effect of culture thickness on the PA phase delay of the bacteria, the phase delay difference between each colony and surrounded culture was measured and the average values are shown in Fig. 3a. According to this bar chart, it is obvious that PA amplitude cannot be used for differentiating these two bacteria. On the contrary, the viscoelasticity ratio of *Staphylococcus aureus* as a Gram-positive bacteria is 1.77 times the value of *Acinetobacter* *baumannii* as a Gram-negative bacteria (22.55° phase delay for *Staphylococcus aureus* and 12.72° phase delay for *Acinetobacter baumannii*). Figure 3b depicts the sine waves of excitation signal as well as the generated PA signals for each bacteria of the second set.

## Discussion

### System performance verification

**Figure 4:**
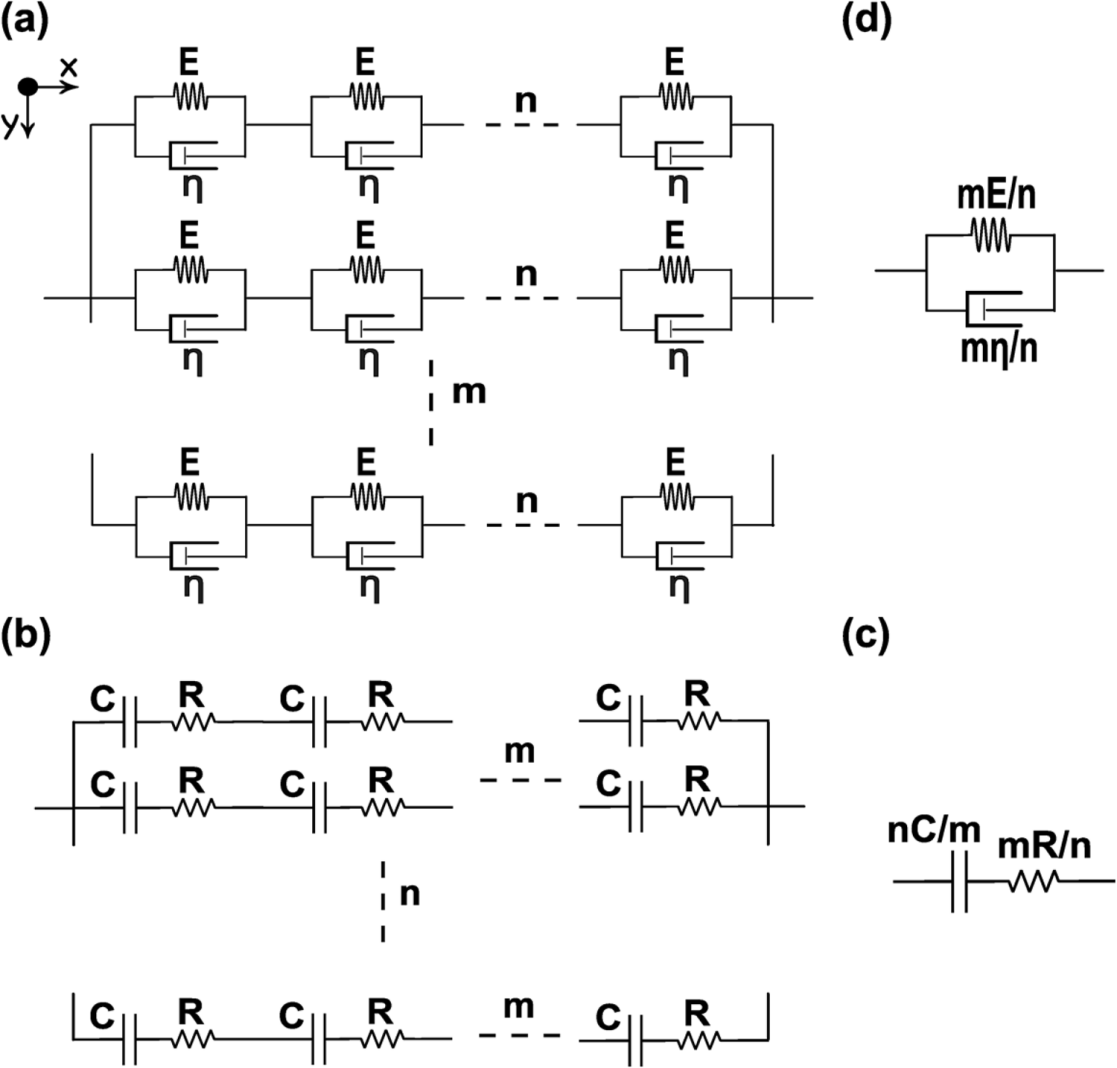
(**a**) Equivalent mechanical system. (**b**) Equivalent electrical circuit. (**c**) Final electrical model. (**d**) Final mechanical model.

Gelatin phantoms and chicken tissues were utilized as the samples to verify the system capabilities by comparing the results provided in the literature. Zhao et al. showed that for gelatin samples with 4% and 7% concentrations, the PA phase delay changed by approximately 22.5%^63^. Based on the results of our experiments, the viscoelasticity ratio of 100 g/L gelatin concentration was changed by 22.8% for the sample with 200 g/L concentration, which is in good correspondence with the previous results^63^. Gao et al. used the liver, fat and muscle of a pig to demonstrate their system output and compared the PA phase delay with rheometer results^68^. They found that liver tissue has the highest viscoelasticity followed by fat and muscle respectively with roughly a 23% variation in the PA phase delay. As represented in Fig. 4, the viscoelasticity ratio of the liver, fat and muscle of a chicken has a similar trend. In addition, muscle tissue had 20% less phase delay compared to liver tissue. The slight difference between our results and the results provided by Gao et al. could be related to the difference between the tissue constituents of pig and chicken and the sample preparation method.

### PA viscoelasticity measurement of bacteria

There are several factors contributing to the viscoelasticity properties of the bacteria including growth conditions as well as specific bacteria properties. As the samples were prepared under controlled conditions with no particular treatments, the growth-related properties were consistent, and the observed differences in viscoelasticity can be attributed solely to the inherent properties of the bacteria themselves. Although the bacterial properties influencing viscoelasticity can be broadly divided into cell envelope structure and biofilm formation, it is not possible to dismiss the potential biofilm formation. Therefore, the different viscoelastic properties could be due to the type of bacteria or the amount of extracellular polymeric substances produced by bacteria. The primary reason for the difference in viscoelasticity between *Acinetobacter baumannii* and *Staphylococcus aureus* is likely the dissimilarities in their cell envelope structures. Previous researches have shown that the cell envelope exhibits similar viscoelastic properties on both the nanoscale and microscopic scale, as measured by AFM^28,30^. Therefore, it is reasonable to expect that these differences in cell envelope structure would also influence the viscoelastic behavior of bacterial colonies, consistent with the behavior observed at the single-cell level. Although the most common mechanical property of the bacteria cells which was reported previously is the equivalent elastic spring constant, Vadillo-Rodriguez et al. investigated the local viscoelastic properties of Gram-positive (*Bacillus subtilis*) and Gram-negative (*Escherichia coli*) bacteria cells by exploiting AFM (which heavily relies on the operator expertise and is not easily scalable) and creep response^30^. They have modelled viscoelasticity with a standard solid model, which describes this mechanical property as an instantaneous elastic response followed by a delayed elastic response, which can be represented as the KV model. Based on their findings, the delayed elastic response of each cell is related to the liquid-like nature of the membrane bilayer having viscose properties. Due to the structural difference between the Gram-positive and Gram-negative bacteria, it is expected that Gram-positive bacteria with only one bilayer membrane buried below a thick peptidoglycan layer have considerably higher viscosity compared to the Gram-negative bacteria composed of two membrane bilayers and a thin peptidoglycan layer. They quantified the characteristic response time as the viscosity-to-elasticity ratio for both bacteria types and demonstrated a higher viscoelasticity ratio for Gram-positive bacteria. In our system, we modeled the viscoelasticity of each cell with the KV model and found a similar trend to the described study^30^. As the size of the laser beam is smaller than a single colony with uniform irradiation, and considering the laser focal length, there is a multi-layered structure of the cells whose mechanical response is measured at each point. In our proposed model, each layer of the cells located in the x–z plane is introduced with one dimension (symmetry assumption) and a number of these layers are stacked over each other on the y-axis. As a result, the mechanical model for the cells in the x-axis is a series connection of the KV models and the equivalent model of the stacked layers is a parallel combination of the KV models. The final model for the bacterial colony is illustrated in Fig. 4a assuming n and m as the number of cells in the x and y axis respectively. By converting the mechanical components into the analogous electrical elements (capacitance value is the inverse of spring constant and resistance is equal to the damping coefficient), the equivalent circuit for the mentioned structure can be depicted as Fig. 4b. By calculating the final values of capacitor and resistor, the spring constant and damping coefficient for a bacterial colony will be determined (${E}_{f}=mE/n, {\eta }_{f}=m\eta /n$). Since the viscoelasticity ratio of the final mechanical model is the same as the value for a single cell, we can expect similar behaviour in the colony scale compared to the single-cell viscoelasticity.

As the samples for this study are the colonies of bacteria located on top of the cultured media, we need to focus the laser on the sample surface to get only the information of the colonies, so the distance between the laser and the sample should be kept constant for all the measurements. Furthermore, in order to improve the system performance and make it insensitive to the thickness of the cultured media, it is better to use hollow shape ultrasound transducer in a reflection mode system in future.

## Methods

### Principle of PAVE

**Figure 5:**
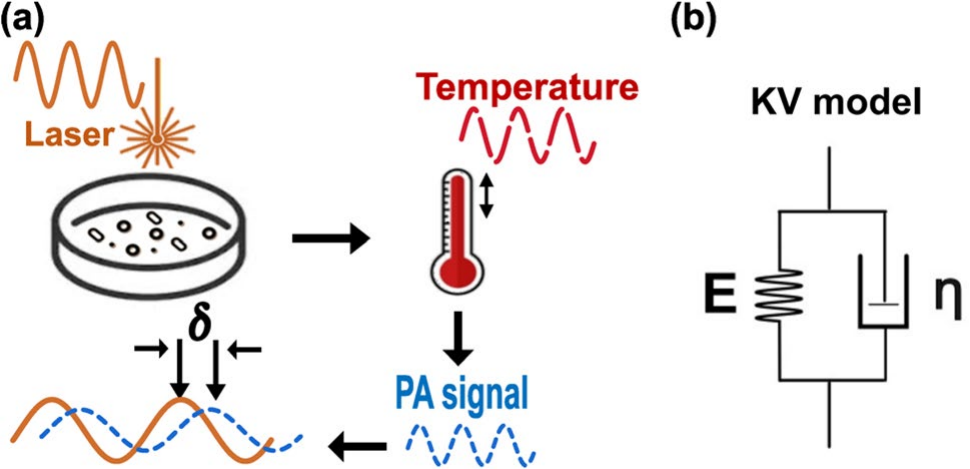
(**a**) PAVE principle. (**b**) Kelvin–Voigt model.

The principle of photoacoustic viscoelasticity is demonstrated in Fig. 5a. The sample is irradiated with an amplitude-modulated laser beam. The light intensity ($I$) is represented in Eq. (1).

1 $$I={\left(1/2\right)I}_{0}\left(1+\mathrm{cos}\omega t\right),$$

where ${I}_{0}$ is time-averaged light intensity, and $\omega$ is the modulation frequency. Light absorption by the sample leads to the temperature fluctuation in the periodic form of $T={T}_{0}{e}^{i\omega t}$ which induces thermal stress due to the thermal expansion. The cyclic thermal stress causes the sinusoidal strain generation in the form of force-produced PA waves with the same frequency of the excitation but with a phase lag owing to the viscoelastic damping effect of the sample. According to the rheological Kelvin-Voigt model (depicted in Fig. 5b), the relation between stress, $\sigma$ and strain, $\varepsilon$ can be expressed by Eq. (2)^69^.

2 $$\sigma \left(t\right)=E\varepsilon \left(t\right)+\eta \dot{\varepsilon }\left(\mathrm{t}\right),$$

where $E$ is Young’s modulus and $\eta$ is the coefficient of viscosity. The Fourier transform of Eq. (2) can be written as Eq. (3) by the periodic assumption of stress and strain.

3 $$\sigma \left(\omega \right)=E\varepsilon \left(\omega \right)+i\omega \eta E\varepsilon \left(\omega \right).$$

The phase lag, $\delta$ between the PA signal (strain) and laser excitation (stress) can be calculated by Eq. (4).

4 $$\delta =\mathrm{arctan}\left(\eta \omega /\mathrm{\rm E}\right).$$

As can be seen in Eq. (4), there is a direct relation between the phase delay and the viscoelasticity ratio of the sample.

### Experimental setup

**Figure 6:**
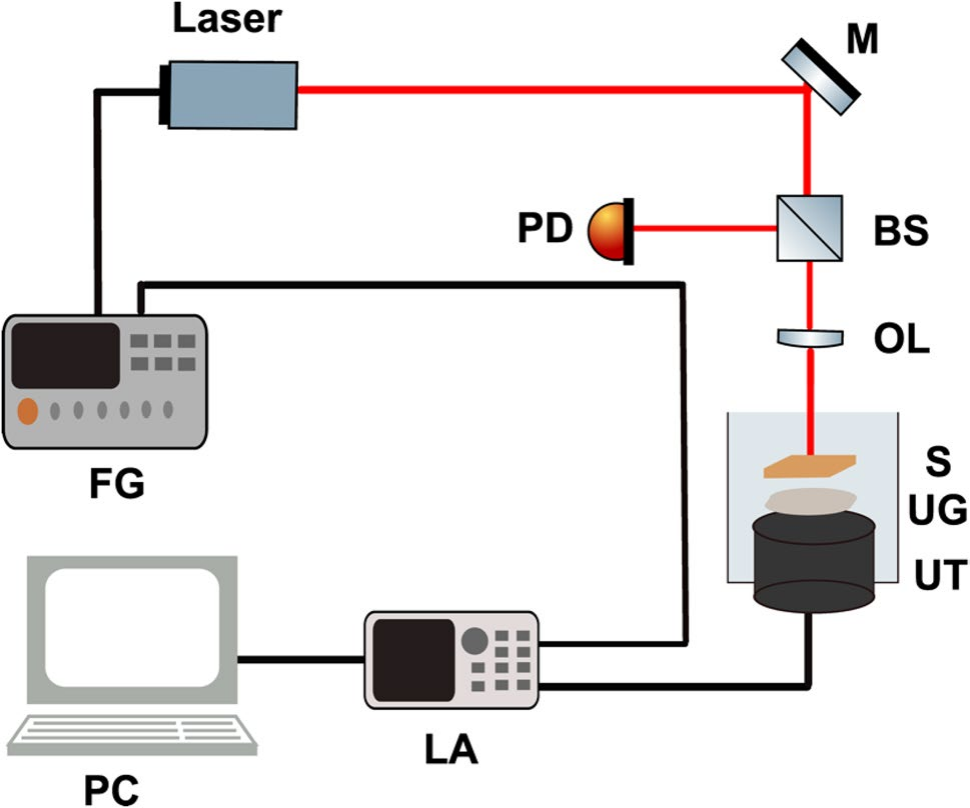
Transmission PAVE system. *FG* function generator, *M* mirror, *BS* beam splitter, *PD* photodiode, *OL* optical lens, *S* sample, *UG* ultrasound gel, *UT* ultrasound transducer, *LA* lock-in amplifier.

A schematic diagram of the transmission mode PAVE system is illustrated in Fig. 6. A customized fibre-coupled CW laser (AKHX248081000D-AL01A, Zhuhai Aike Photonics Technology Ltd) with 808 nm wavelength and maximum output power of 1 W was used as the excitation source. The laser beam was modulated sinusoidally with the frequency of 50 kHz by the laser driver and focused by an adjustable lens on the sample (the diameter of the laser spot was 0.8 mm meaning that the laser fluence was well within the ANSI limit). An ultrasonic transducer (DYW-50 kHz, Dayu Electric) with a center frequency and diameter of 50 kHz and 63 mm collected the produced PAVE signal. The signal was then fed into a homemade preamplifier and a homemade two-channel lock-in amplifier to calculate both the amplitude ratio and phase lag between the excitation source and the detected signal. The measured phase delay consists of the system phase delay (due to the electronic system), relaxation time for non-radiative transition and the viscoelasticity-related phase delay. As the operating frequency of system is fixed (50 kHz), the phase delay of the electronic system is fixed during the measurement. The order of time delay for non-radiative transition is about ${10}^{-11}\mathrm{ s}$ which can be ignored comparing with the modulation frequency 50 kHz (T = 20 µs). Therefore, the measured phase delay can be regarded for comparing the viscoelasticity of the samples^68^. Finally, the signal was digitized by a DAQ card (USB-4716 Advantech) and imported into the computer. The software link with the board and further processing were performed using the MATLAB platform. Compared to the previously reported systems for PAVE, the major difference is the method of modulation of the excitation laser. We have employed electronic laser beam modulation which provides higher speed modulation, flexible modulation formats, wider bandwidths, compactness and reliability in comparison to the electro-optic modulators (used in previous methods).

### Phantom and bacterial samples preparation

- Gelatin and chicken samples

To mimic the mechanical properties of biological samples and verify the system capability, gelatin phantoms were prepared by controlling the gelatin-water mixture. Gelatin-water mixtures with the concentration of 100 and 200 g/L were heated in a water bath to melt the mixture and then, poured into the molds. Moreover, chicken's muscle, fat and liver have been chosen to evaluate the system performance and compare the results with previously reported values in the literature.

- Bacterial strains and growth conditions

*Staphylococcus aureus* ATCC 12600 and *Acinetobacter baumannii* ATCC BAA-747 were provided by the microbiology department of Shahid Beheshti University of Medical Sciences. The bacteria samples had been kept at – 70 °C in Tryptic Soy Broth (TSB) culture medium with glycerol. To perform the test, bacteria were defrosted and cultured on Luria Bertani (LB) agar (HiMedia, India) medium at 37 °C for 18 h. In order to increase the accuracy and precision of the tests, culture media with similar conditions and thickness were prepared. Right after the growth of bacteria, separate colonies of each bacterium were cut along with the culture medium and examined.

## Conclusions

In summary, to the best of our knowledge, the viscoelasticity investigation of *Acinetobacter baumannii* as Gram-negative bacteria and *Staphylococcus aureus* as Gram-positive bacteria in a colony scale based on the PA signal generation is proposed for the first time. The results showed that Gram-positive bacteria have higher phase delay or viscoelasticity compared to Gram-negative bacteria (22.55° vs 12.72° phase delay) due to the structural difference between the envelope of Gram-negative and Gram-positive bacteria. PAVE is a cost-effective, reliable technique with simple sample preparation in comparison to AFM as an operator-dependent method; further studies would expand and discover the capacity of viscoelasticity measurement by the PA method accordingly in the field of biology. Meanwhile, a mechanical model has been also introduced for a colony of bacteria.

## Data Availability

All data generated or analysed during this study are available from the corresponding author on request.

## References

[1] Darling EM, di Carlo D (2015). High-throughput assessment of cellular mechanical properties. Annu. Rev. Biomed. Eng..

[2] Moeendarbary E, Harris AR (2014). Cell mechanics: Principles, practices, and prospects. Wiley Interdiscip. Rev. Syst. Biol. Med..

[3] Puig-de-Morales-Marinkovic M, Turner KT, Butler JP, Fredberg JJ, Suresh S (2007). Viscoelasticity of the human red blood cell. Am. J. Physiol. Cell Physiol..

[4] Levental I, Georges PC, Janmey PA (2007). Soft biological materials and their impact on cell function. Soft Matter.

[5] Vadillo-Rodríguez V, Dutcher JR (2011). Viscoelasticity of the bacterial cell envelope. Soft Matter.

[6] Marx A, Pless J, Mandelkow E-M, Mandelkow E (2000). On the rigidity of the cytoskeleton. Cell Mol. Biol..

[7] Mitchison J, Swann MM (1954). The mechanical properties of the cell surface. J. Exp. Biol.

[8] Doetsch RN, Cook TM (2012). Introduction to Bacteria and Their Ecobiology.

[9] Baron, S. (ed). *Medical Microbiology*, 4th ed (University of Texas Medical Branch at Galveston, 1996). 21413252

[10] Silhavy TJ, Kahne D, Walker S (2010). The bacterial cell envelope. Cold Spring Harb. Perspect. Biol..

[11] Rohde M (2019). The Gram-positive bacterial cell wall. Microbiol. Spectr..

[12] Costerton JW, Ingram JM, Cheng KJ (1974). Structure and function of the cell envelope of gram-negative bacteria. Bacteriol. Rev..

[13] Mills JP, Qie L, Dao M, Lim CT, Suresh S (2004). Nonlinear elastic and viscoelastic deformation of the human red blood cell with optical tweezers. Mol. Cell. Biomech..

[14] Ayala YA (2016). Rheological properties of cells measured by optical tweezers. BMC Biophys..

[15] Lyubin EV, Khokhlova MD, Skryabina MN, Fedyanin AA (2012). Cellular viscoelasticity probed by active rheology in optical tweezers. J. Biomed. Opt..

[16] Wei M-T (2008). A comparative study of living cell micromechanical properties by oscillatory optical tweezers. Opt. Express.

[17] Lim HG (2020). Investigation of cell mechanics using single-beam acoustic tweezers as a versatile tool for the diagnosis and treatment of highly invasive breast cancer cell lines: An in vitro study. Microsyst. Nanoeng..

[18] Bausch AR, Möller W, Sackmann E (1999). Measurement of local viscoelasticity and forces in living cells by magnetic tweezers. Biophys. J..

[19] Kilinc D, Lee GU (2014). Advances in magnetic tweezers for single molecule and cell biophysics. Integr. Biol..

[20] Shojaei-Baghini E, Zheng Y, Sun Y (2013). Automated micropipette aspiration of single cells. Ann. Biomed. Eng..

[21] Zhou EH, Lim CT, Quek ST (2005). Finite element simulation of the micropipette aspiration of a living cell undergoing large viscoelastic deformation. Mech. Adv. Mater. Struct..

[22] Yang C, Chen D, Hong X (2016). Estimation of viscoelastic properties of cells using acoustic tweezing cytometry. J. Ultrasound Med..

[23] Serhatlioglu M, Asghari M, Tahsin Guler M, Elbuken C (2019). Impedance-based viscoelastic flow cytometry. Electrophoresis.

[24] Gerum R (2022). Viscoelastic properties of suspended cells measured with shear flow deformation cytometry. Elife.

[25] Laurent VM (2002). Assessment of mechanical properties of adherent living cells by bead micromanipulation: Comparison of magnetic twisting cytometry vs optical tweezers. J. Biomech. Eng..

[26] Rother J, Nöding H, Mey I, Janshoff A (2014). Atomic force microscopy-based microrheology reveals significant differences in the viscoelastic response between malign and benign cell lines. Open Biol..

[27] Wang Y (2016). Quantitative analysis of the cell-surface roughness and viscoelasticity for breast cancer cells discrimination using atomic force microscopy. Scanning.

[28] Vadillo-Rodriguez V, Beveridge TJ, Dutcher JR (2008). Surface viscoelasticity of individual gram-negative bacterial cells measured using atomic force microscopy. J. Bacteriol..

[29] Efremov YM, Okajima T, Raman A (2020). Measuring viscoelasticity of soft biological samples using atomic force microscopy. Soft Matter.

[30] Vadillo-Rodriguez V, Schooling SR, Dutcher JR (2009). In situ characterization of differences in the viscoelastic response of individual gram-negative and gram-positive bacterial cells. J. Bacteriol..

[31] Huang W (2019). In vivo quantitative photoacoustic diagnosis of gastric and intestinal dysfunctions with a broad pH-responsive sensor. ACS Nano.

[32] Ye J (2020). Quantitative photoacoustic diagnosis and precise treatment of inflammation in vivo using activatable theranostic nanoprobe. Adv. Funct. Mater..

[33] Sim C (2015). Photoacoustic-based nanomedicine for cancer diagnosis and therapy. J. Control. Release.

[34] Zeng Y, Dou T, Ma L, Ma J (2022). Biomedical photoacoustic imaging for molecular detection and disease diagnosis: “Always-On” and “Turn-On” probes. Adv. Sci..

[35] Jin Y, Yin Y, Li C, Liu H, Shi J (2022). Non-invasive monitoring of human health by photoacoustic spectroscopy. Sensors.

[36] Cao F, Qiu Z, Li H, Lai P (2017). Photoacoustic imaging in oxygen detection. Appl. Sci..

[37] Zhang D (2021). Photoacoustic imaging of in vivo hemodynamic responses to sodium nitroprusside. J. Biophotonics..

[38] Li M, Tang Y, Yao J (2018). Photoacoustic tomography of blood oxygenation: A mini review. Photoacoustics.

[39] Zhang Y, Yu J, Kahkoska AR, Gu Z (2017). Photoacoustic drug delivery. Sensors.

[40] Xia J, Kim C, Lovell FJ (2015). Opportunities for photoacoustic-guided drug delivery. Curr. Drug Targets.

[41] Park B, Park S, Kim J, Kim C (2022). Listening to drug delivery and responses via photoacoustic imaging. Adv. Drug Deliv. Rev..

[42] Moore C, Chen F, Wang J, Jokerst JV (2019). Listening for the therapeutic window: Advances in drug delivery utilizing photoacoustic imaging. Adv. Drug Deliv. Rev..

[43] Moore C, Jokerst JV (2019). Strategies for image-guided therapy, surgery, and drug delivery using photoacoustic imaging. Theranostics.

[44] Lee CY (2020). Photoacoustic imaging to localize indeterminate pulmonary nodules: A preclinical study. PLoS ONE.

[45] Wang Y (2017). Preclinical evaluation of photoacoustic imaging as a novel noninvasive approach to detect an orthopaedic implant infection. JAAOS J. Am. Acad. Orthop. Surg..

[46] Gorey A (2019). Differentiation of malignant from benign thyroid nodules using photoacoustic spectral response: A preclinical study. Biomed. Phys. Eng. Express..

[47] Valluru KS, Wilson KE, Willmann JK (2016). Photoacoustic imaging in oncology: Translational preclinical and early clinical experience. Radiology.

[48] Gargiulo S, Albanese S, Mancini M (2019). State-of-the-art preclinical photoacoustic imaging in oncology: Recent advances in cancer theranostics. Contrast Media Mol. Imaging.

[49] Menyaev YA (2016). Preclinical photoacoustic models: Application for ultrasensitive single cell malaria diagnosis in large vein and artery. Biomed. Opt. Express.

[50] Park S, Lee C, Kim J, Kim C (2014). Acoustic resolution photoacoustic microscopy. Biomed. Eng. Lett..

[51] Jeon S, Kim J, Lee D, Baik JW, Kim C (2019). Review on practical photoacoustic microscopy. Photoacoustics.

[52] Periyasamy V, Das N, Sharma A, Pramanik M (2019). 1064 nm acoustic resolution photoacoustic microscopy. J. Biophotonics..

[53] Hai P, Yao J, Maslov KI, Zhou Y, Wang LV (2014). Near-infrared optical-resolution photoacoustic microscopy. Opt. Lett..

[54] Xia J, Yao J, Wang LV (2014). Photoacoustic tomography: Principles and advances. Progr. Electromagn. Res..

[55] Jiang, H. *Photoacoustic Tomography* (CRC Press, 2018).

[56] Wang LV (2008). Prospects of photoacoustic tomography. Med. Phys..

[57] Wang LV, Yao J (2016). A practical guide to photoacoustic tomography in the life sciences. Nat. Methods.

[58] Guo H, Li Y, Qi W, Xi L (2020). Photoacoustic endoscopy: A progress review. J. Biophotonics..

[59] Yang J-M (2009). Photoacoustic endoscopy. Opt. Lett..

[60] Yoon T-J, Cho Y-S (2013). Recent advances in photoacoustic endoscopy. World J. Gastrointest. Endosc..

[61] Xiong K, Yang S, Li X, Xing D (2018). Autofocusing optical-resolution photoacoustic endoscopy. Opt. Lett..

[62] Qu Y (2018). Transvaginal fast-scanning optical-resolution photoacoustic endoscopy. J. Biomed. Opt..

[63] Zhao Y, Yang S, Chen C, Xing D (2014). Simultaneous optical absorption and viscoelasticity imaging based on photoacoustic lock-in measurement. Opt. Lett..

[64] Zhao Y, Chen C, Yang S, Xing D (2016). Mechanical evaluation of lipid accumulation in atherosclerotic tissues by photoacoustic viscoelasticity imaging. Opt. Lett..

[65] Wang Q, Shi Y, Yang F, Yang S (2018). Quantitative photoacoustic elasticity and viscosity imaging for cirrhosis detection. Appl. Phys. Lett..

[66] Jin D, Yang F, Chen Z, Yang S, Xing D (2017). Biomechanical and morphological multi-parameter photoacoustic endoscope for identification of early esophageal disease. Appl. Phys. Lett..

[67] Wang Q, Shi Y (2020). Photoacoustic viscoelasticity imaging for the detection of acute hepatitis: A feasibility study. Biophys. Rep..

[68] Gao G, Yang S, Xing D (2011). Viscoelasticity imaging of biological tissues with phase-resolved photoacoustic measurement. Opt. Lett..

[69] Zhao Y, Yang S (2013). Photoacoustic viscoelasticity imaging of biological tissues with intensity-modulated continuous-wave laser. J. Innov. Opt. Health Sci..

